# Examining the impact of self-stigma on workplace well-being: an empirical investigation of medical students with physical disabilities in China and the moderating role of trait mindfulness

**DOI:** 10.1186/s12909-024-05554-4

**Published:** 2024-07-09

**Authors:** Minqiao Hu, Xiongfu Wu, Shuang Qiu

**Affiliations:** 1 Business School, Sichuan University, Chengdu, China; 2 School of Foreign Languages, Chengdu University of Traditional Chinese Medicine, Chengdu, China

**Keywords:** Workplace well-being, Self-stigma, Trait mindfulness, Medical students with physical disabilities, Cognitive consistency theory

## Abstract

**Background:**

As societal evolution unfolds in China, individuals with physical disabilities are increasingly provided opportunities in higher education, particularly in the field of medicine. However, these medical students often encounter bias in their work environments, including during internships, which fosters self-stigma and impedes their experience for workplace well-being (WWB). Such a decrease in WWB detrimentally affects not only their mental health in the workplace but also hinders their sense of personal worth and assimilation into broader society. This study aims to examine the challenges faced by medical students with physical disabilities in China as they aspire to achieve WWB, and to explore potential intervention strategies.

**Methods:**

Leveraging cognitive consistency theory (CCT), we introduces a conceptual framework to examine the relationships among self-stigma, perceived discrimination, and WWB. It also investigates the role of trait mindfulness as a potential mitigating factor in this dynamic. We employed the Internalized Stigma of Mental Illness Scale (ISMIS), Discrimination Perception Questionnaire (DPQ), Workplace Well-being Subscale (WWBS), and Mindful Attention Awareness Scale (MAAS) to survey 316 medical students with physical disabilities. Statistical analyses, including correlation, regression, and moderated mediation effect assessments, were conducted using SPSS 22.0 and AMOS 24.0.

**Results:**

A notable negative correlation exists between self-stigma and WWB (*r* = -0.56, *p* < 0.01). Perceived discrimination partially mediates the relationship between self-stigma and WWB. The direct effect of self-stigma and its mediating effect through perceived discrimination account for 60.71% and 21.43% of the total effect, respectively. Trait mindfulness moderates the latter part of this mediating pathway. Moderation models indicate that trait mindfulness has a significant negative moderating effect on the impact of perceived discrimination on WWB (β = -0.10, *p* < 0.001).

**Conclusions:**

Self-stigma adversely affects the positive work experiences of medical students with physical disabilities by eliciting a heightened sensitivity to discriminatory cues, thereby undermining their WWB. Trait mindfulness can effectively counter the detrimental effects of perceived discrimination on WWB. Consequently, this study advocates for the systematic incorporation of mindfulness training into educational services and workplace enhancement programs for medical students with disabilities, aiming to foster an inclusive and supportive external environment.

## Background

Over the past four decades, Chinese higher education institutions have significantly diversified the academic disciplines available to students with physical disabilities. The medical field has become a popular choice among these students [1], who form a unique group within the disabled population. For medical colleges and healthcare institutions, it is crucial to ensure that these students’ medical skills and behavioral tendencies meet the professional standards required in medicine. Another critical objective is to provide psychological and quality-oriented education that facilitates their successful integration into mainstream society.

Work is an essential aspect of personal life and a marker of social integration. For medical students with physical disabilities, work is not only a livelihood but also a vital means of forming relationships, enhancing social status, and achieving self-worth [2]. Recent shifts in academic focus from a pathological to a strengths-based perspective in positive psychology have heightened interest in the well-being of disabled individuals. Although most research has concentrated on their subjective well-being (SWB) [3, 4], assessing life satisfaction broadly, there is a scarcity of studies on the well-being of disabled individuals in workplace settings.

Medical workers are typically expected to be flawless, yet medical students with physical disabilities often face prejudice and negative attitudes, including during internships [5].These challenges extend beyond the acquisition of professional skills, as these students must also navigate societal judgments and may internalize negative perceptions, leading to self-stigma [6]. Such experiences can heighten perceptions of discrimination and result in adverse cognitive outcomes. The Cognitive Consistency Theory (CCT) posits that humans inherently strive for cognitive alignment [7], where cognitive congruence is achieved by aligning factual information with expectations [8, 9]. The greater the discrepancy between expectations and reality, the more intense the negative experiences are likely to be. For medical students with physical disabilities, their inherently optimistic view of their professional future represents ‘expectation,’ while the adverse cognitive outcomes stemming from perceived discrimination constitute ‘factual information.’ Cognitive dissonance may occur when there is a misalignment, adversely affecting their well-being at workplace.

WWB is a relatively stable psychological state, influenced by individual characteristics [10]. Thus, incorporating variables related to individual traits may alleviate the effects of perceived discrimination on WWB. Mindfulness, celebrated for its fundamental principle of being present and non-judgmental, has received considerable attention in psychotherapy research [11]. Nonetheless, it is essential to acknowledge that, although existing literature supports mindfulness in facilitating introspection and mitigating negative emotional states, there is a gap regarding how individuals experiencing high levels of discrimination can diminish excessive self-focus to enhance WWB. Vago and Silbersweig [12] suggest that trait mindfulness may act as a metacognitive strategy, promoting a transition from self-enhancement to self-transcendence, indicating its potential in correcting adverse cognitive impacts stemming from self-preoccupation. Moreover, recent studies have shown that trait mindfulness helps individuals escape from excessive self-rumination and fosters the development of virtues characterized by nobility and universality [13, 14], which could reshape expectations towards positive work experiences.

**Fig. 1:**
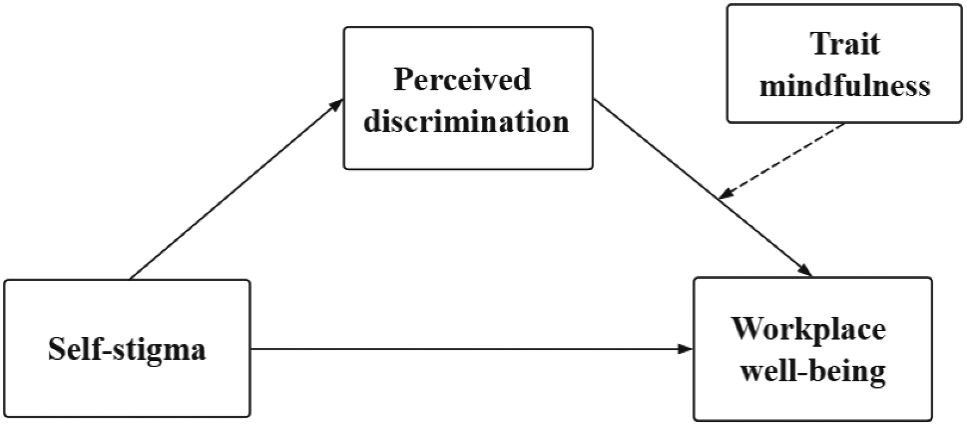
The proposed model

In summary, guided by the CCT, this study aims to explore the mechanism through which self-stigma affects WWB among medical students with physical disabilities. It also seeks to investigate the moderating effect of trait mindfulness on this relationship. This research focuses on this specific demographic and proposes a moderated mediation model as illustrated in Fig. 1, formulating the following hypotheses:

***H1: Self-stigma is negatively related to WWB among medical students with physical disabilities.***

***H2: The relationship between self-stigma and WWB among medical students with physical disabilities is mediated by perceived discrimination.***

***H3: Trait mindfulness moderates the relationship between perceived discrimination and WWB. The detrimental impact of perceived discrimination on WWB is less pronounced for medical students with physical disabilities with higher trait mindfulness compared to those with lower levels.***

## Theory and hypotheses development 

Therefore, we predict a similar relationship between self-stigma and WWB. We put forward the following hypothesis: H1: Self-stigma is negatively related to WWB among medical students with physical disabilities.

### Self-stigma and workplace well-being (WWB)

Stigma refers to the negative stereotype outcome of demonizing, condemning, and excluding specific groups from society [15]. Thornicroft et al. [16] posit that stigma frequently stems from misinformed or biased perceptions of mental and physical disabilities. Individuals with physical challenges often find themselves at the receiving end of such stigma. Societal biases can make these individuals less accepting of their own physical limitations. Consequently, they might internalize the derogatory labels ascribed by the larger society, culminating in *Self-stigma* [17]. The self-stigmatization makes this demographic recognize, resonate with, and ultimately, adopt the negative stereotypes propagated by external world [18]. Studies have underscored that self-stigma exacerbates psychological distress and self-deprecation, erodes self-worth and social identity, and is linked with reduced self-esteem, resilience, and cognitive aptitude [19].

Warr [20] proposed the concept of W*WB*, focusing on studying well-being in the context of work. Currently, there is no consensus among scholars regarding the specific definition of WWB; however, it is generally agreed that it pertains to individuals’ perceived experiences with their current work and represents a particular aspect of employee well-being [21]. Specifically, WWB, in contrast to occupational well-being, which encompasses the entire career trajectory of individuals [22], places emphasis on individuals’ current work experience. It reflects the comprehensive evaluation of the degree of subjective delight an individual derives from their current work experience, taking into account social, emotional, physical, and monetary factors [23], involving complexity and variability in individuals’ cognitive emotions and emotional experiences at their current work [24]. Building upon theoretical frameworks such as person-environment fit [25], the job demands-resources model [26], and the employee performance-well-being model [27], existing literature has sorted out the formation mechanism of WWB while clarifying how labor (work) can contribute to experiencing well-being.

However, medical students with physical disabilities encounter intricate and insidious prejudice at work, such as an excessive display of sympathy from leaders, contemptuous treatment from colleagues [28], and even implicit rejection by patients. While internalizing these biases, individuals may overlook their inherent abilities and potential, leading to self-negation or reduced confidence in planning and realizing work objectives [29, 30]. Individuals’ motivation to acquire and sustain work-associated value also declines [31], posing challenges to experiencing WWB. From this perspective, self-stigma could lead to cognitive dissonance among individuals.

The CCT posits that beliefs contrary to one’s expectations, often referred to as ‘bad news,’ impair the achievement of desired outcomes and amplify negative emotions, subsequently hindering well-being [7]. For medical students with physical disabilities, their sense of self-worth often stems from their current working roles. Despite their disabilities, they perceive themselves as valuable contributors to organizations and society at large [32]. Nonetheless, self-stigmatization induces the absorption of external stereotypes, biases, and discriminatory views, making them see these as ‘bad news’ that they deserve. Such ‘bad news’ cause these students to confront the ‘factual information’ that their personal worth, professional competence and contribution have been devalued because of their physical limitations [33]. This contradicts their ‘expectations’ that current work can return positive emotional experiences such as social recognition and self-affirmation. As a result, it leads to uncomfortable cognitive dissonance and a decline in WWB. Moreover, a empirical study on lesbians and gays has also revealed a adverse association between individual stigma consciousness and well-being [34].

### Perceived discrimination as a mediator

Discrimination entails the prejudicial treatment or differentiation of specific groups based on characteristics such as ethnicity, gender, nationality, disability, among others. Such treatment often leads to their marginalization and social exclusion, affecting the way the external world behaves towards them and restricting their opportunities and rights. Experiencing discrimination considerably influences an individual’s self-esteem and psychological well-being [35], and can also undermine the quality of interpersonal relationships, fostering animosity between different groups [36]. *Perceived discrimination* denotes an individual’s interpretation of this prejudicial treatment, evidenced in their recognition of overt behavioral acts, dismissive attitudes, or particular biased societal structures [37].

While existing research suggests that individuals without disabilities may not consistently hold adverse perceptions of those with physical challenges [38], disabled individuals often experience feelings of discrimination. Corrigan et al. [39] suggest a potential correlation with self-stigma, which can erode individual protective resources such as self-esteem, self-efficacy and perceived competence, thus affecting cognitive functioning [40]. The developmental model of discrimination perception posits that cognitive factors serve as the foundation for perceived discrimination, accounting for individual variances therein [41]. In some instances, cognitive elements alone can determine the level of discrimination one perceives [42]. Importantly, disabled individuals who internalize stigma may amplify their awareness of and sensitivity to discriminatory cues, leading to heightened discrimination perception [43]. Consequently, subtle prejudice, even imagined instances, may be perceived and accentuated as overt discrimination. This heightened perception, as suggested by the CCT, emerges as individuals strive to maintain cognitive equilibrium.

Work should serve as one of the primary means for adults to achieve individual purpose and social integration [44]. However, medical students with physical disabilities face more significant challenges in gaining WWB compared to their non-disabled peers. Perceived discrimination often undermines their joy of participation and fulfillment, as well as their self-worth and self-efficacy. For example, in focus group interviews, employees with disabilities expressed feelings of being treated as ‘second-class citizens’ and believed that their intelligence and involvement were greatly underestimated [45]. Moreover, multiple studies have shown that individuals with disabilities report perceiving lower-quality work experiences, which are characterized by reduced earnings, limited training, and fewer benefits [46]. These disparities are not limited to tangible aspects of the job alone. Individuals, even with minor physical limitations, often feel less supported or recognized by their colleagues and superiors compared to other [47]. Moreover, strained relationships, disrespectful treatments, and obstructed opportunities add to work-related stress, which could further deteriorate their well-being at workplace [48]. Building on the CCT, the perception of discrimination means that medical students with physical disabilities experience negative cognitive reactions like self-doubt, self-discrimination, and self-pity. This disrupts their expectations that work should enable them to achieve objectives and integrate into society, impairing WWB. The following mediating hypothesis is thus proposed: H2: The relationship between self-stigma and WWB among medical students with physical disabilities is mediated by perceived discrimination

### Trait mindfulness as a moderator

Originating from ancient Eastern meditation traditions, mindfulness has secured a position in the study of contemporary psychology. According to Shonin et al. [49], mindfulness can manifest either as a consistent trait (‘*Trait Mindfulness’*) or as a state of being (‘State Mindfulness’). While the presence of trait mindfulness naturally varies among individuals, state mindfulness typically emerges or is sustained following mindfulness training interventions. This study explores the trait mindfulness of medical students with physical disabilities, especially considering their limited access to tailored mindfulness training. Trait mindfulness is not static; it is shaped by both nature and nurture. Consequently, the efficacy of trait mindfulness can be enhanced through specific educational and therapeutic programs. Current literature emphasizes the significance of trait mindfulness in addressing a variety of mental health challenges. For instance, its presence often correlates with improved well-being [50].

The current body of literature identifies two central aspects of trait mindfulness: awareness and sustained attention. First, the metacognitive awareness, intrinsic to mindfulness, strengthens cognitive flexibility and adaptability, thereby suppressing unconscious and habitual impulses [51]. This heightened sense of awareness helps individuals recognize and understand negative emotions in the workplace. Second, mindfulness involves deliberate acknowledgment of the present experience, enhancing one’s ability to sustain attention, characterized by stability, control, and effective concentration [52]. The Cognitive Consistency Theory (CCT) posits that individuals experience discomfort and stress when they encounter cognitive dissonance, that is, the presence of inconsistent cognitive elements [53]. Medical students with physical disabilities experience distress not only from external stimuli but also from their subjective interpretation and rumination of these stimuli, which can be referred to as their cognitive reactions. When perceived discrimination exacerbates their attention to negative cognitive reactions, trait mindfulness emerges as a meta-awareness intervention. This intervention promotes mental expansiveness and ‘decentering,’ which reduce judgmental self-narratives and counterproductive rumination, thereby balancing attentional bias [54].

Additionally, the buffering effect of trait mindfulness on attentional bias is also rooted in an individual’s shift from self-improvement to altruistic transcendence. This transition aligns with the S-ART model, which includes self-awareness, self-regulation, and self-transcendence, and is based on the neurobiology of mindfulness [12]. Mindful self-awareness reduces attentional biases towards subtle discrimination and excessive self-referentiality, fostering self-regulation, strengthening connections with colleagues, organizations, and external society at large. By moving beyond narrow self-concerns and gaining a profound understanding of one’s inherent worth and behavioral patterns, individuals cultivate a broader perspective on the meaning of life and virtue [14]. This perspective reframes the expectation that ‘work contributes to well-being.’ Based on above arguments, we suggest that trait mindfulness will function as a protective factor against the adverse effects of perceived discrimination on WWB. H3: Trait mindfulness moderates the relationship between perceived discrimination and WWB. The detrimental impact of perceived discrimination on WWB is less pronounced for medical students with physical disabilities with higher trait mindfulness compared to those with lower levels.

## Methods

### Study sample

The study secured approval from the Ethics Committee of Chengdu University of Traditional Chinese Medicine. An pilot study was conducted to validate the questionnaire, wherein 25 valid questionnaires were accumulated, resulting in a response rate of 83.33%. Subsequent to comprehensive statistical analysis, modifications were made to certain aspects of the questionnaire, thereby initiating the main survey. In collaboration with 22 medical institutions and 4 medical colleges in Sichuan province, we recruited disabled medical students who expressed interest in our study. The recruit criterion were as follows: (1) individuals with physical disabilities complying with the ‘2023 China Practical Assessment Standards for Disabled Persons’ (i.e., excluding visual, auditory, speech, intellectual, and mental disabilities); (2) adults aged 18 and above; (3) undergoing or have completed a medical internship for graduation. Ultimately, 412 medical students on a five-year baccalaureate program satisfying the criteria were recruited for the study. In order to cultivate an environment in which participants could articulately express their perspectives, a detailed letter emphasizing data confidentiality and assuring adherence to academic ethics was provided.

Data collection was conducted over the course of one month in two stages to attenuate the impact of potential common method bias. Two trained staff members provided consistent guidance to ensure respondents completed the questionnaires accurately, reflecting their actual situations. The average response time for respondents to complete the questionnaire ranges from 30 to 40 min. The staff reviewed the questionnaires for validity and discarded any that displayed uniform responses or a wave-like pattern in choices. In the initial stage, data related to self-stigma, perceived discrimination, and control variables (e.g., gender, age, and medical internship duration) were collected. Of the distributed questionnaires, 353 were returned, achieving an 85.68% response rate. Two weeks later, data concerning trait mindfulness and WWB were solicited from those who completed the first wave of surveys. In gratitude for their contribution, participants who successfully completed the surveys were awarded a 30 RMB book voucher. Upon excluding incomplete or patterned responses, 316 valid questionnaires were retained for further analysis, exhibiting a retention rate of 89.52%.The valid sample comprised 164 females (51.90%) and 152 males (48.10%), with a mean age of 22.58 years and an average medical internship duration of approximately 10.67 months (in the final stage of a 12-month medical internship). Their primary specialties included traditional Chinese medicine, acupuncture and massage, internal medicine, rehabilitation medicine and integrated traditional Chinese and Western clinical medicine.

### Measures

#### Self-stigma

The present study utilized a revised Chinese version of the Internalized Stigma of Mental Illness Scale (ISMIS) to measure self-stigma. This version was adapted by Qi [55] from the original scale by Ritsher et al. [56]. The revision underwent rigorous translation-back-translation and group discussion procedures to ensure both cultural and content equivalence with the original scale. This makes it suitable for investigating self-stigma individuals with disabilities [55]. This scale includes four dimensions: depreciation-discrimination, alienation, social avoidance, and stigma resistance, encompassing a total of 23 items. Participants rated each item on a 5-point Likert scale, ranging from 1 (strongly disagree) to 5 (strongly agree), with five items being reverse-scored. Total scores and sub-scale scores were obtained by averaging responses, where higher scores indicated greater self-stigma. The scale’s reliability analysis in the current study yielded a Cronbach’s α coefficient of 0.89.

#### Perceived discrimination

The present study utilized the Discrimination Perception Questionnaire (DDPQ), developed by Li [57], to evaluate participants’ perception of discrimination. This questionnaire consists of 10 items that are rated on a 5-point Likert scale ranging from 1 (strongly disagree) to 5 (strongly agree). For example, one sample item states “During social activities, I perceive that individuals around me demonstrate hesitancy in communication and avoid engaging with me.” The total score for this scale is computed as the sum of individual item scores, with higher scores indicating an elevated perception of encountered discrimination. The scale’s reliability analysis in the current study yielded a Cronbach’s α coefficient of 0.92.

### Trait mindfulness

The present study employed the Mindful Attention Awareness Scale (MAAS), developed by Brown and Ryan [50], to assess participants’ levels of trait mindfulness. This scale has been widely utilized in management research to measure individuals’ attention and awareness of daily life events, particularly among those without prior mindfulness training. An example item was “ I find myself doing things without paying attention.” Participants were instructed to select the description that best reflected their actual experiences for each item. Items were rated on a 5-point Likert scale ranging from 1 (strongly disagree) to 5 (strongly agree), with all 15 items reverse-scored. The scale has been extensively used in studies involving Chinese populations and has demonstrated robust psychometric properties [58]. The scale’s reliability analysis in the current study yielded a Cronbach’s α coefficient of 0.88.

### Workplace well-being

The measurement of WWB was assessed using the Workplace Well-being Sub-scale (WWBS) derived from the Employee Well-being Scale (EWBS) developed by Zheng et al. [24]. The WWB scale consists of six items, such as “I derive genuine enjoyment from my work.” A 5-point Likert scale ranging from 1 (strongly disagree) to 5 (strongly agree) was utilized to assess all items. This scale was specifically designed for studying Chinese individuals within the eastern organizational context and has been shown to have good test validity [59]. The scale’s reliability analysis in the current study yielded a Cronbach’s α coefficient of 0.88.

### Control variables

The demographic variables, including gender, age, and tenure (i.e., duration of medical internship), were incorporated as control variables in order to control for their potential influence on WWB [60].

The data entry, processing, and analyses, including correlation analysis, regression analysis, as well as testing for moderation and mediation effects, were performed using SPSS 22.0 and AMOS 24.0.

## Results

### Common method bias test

The associations between variables were investigated in this research using a questionnaire survey. Considering the potential for common method bias, an exploratory factor analysis was conducted on all variable items using the Harman One-factor Test, with a benchmark value set at 40%. If the variance explained by the first factor prior to rotation exceeds 40%, it would suggest the presence of common method bias. The data results indicate that there are 12 factors with eigenvalues greater than 1, and the contribution of the first factor is 30.38%, which falls below the predetermined threshold of 40%. Therefore, these findings suggest that there is no significant presence of common method bias in this research.

### Descriptive statistics

Table 1 presents the descriptive statistics and correlations for the study variables. The findings highlight a significant positive correlation between self-stigma and perceived discrimination (*r* = 0.51, *p* < 0.001). Conversely, there are negative correlations observed between self-stigma with both WWB (*r* = -0.57, *p* < 0.01) and trait mindfulness (*r* = -0.26, *p* < 0.001). Additionally, WWB shows a negative correlation with perceived discrimination (*r* = -0.56, *p* < 0.01) but a positive one with trait mindfulness (*r* = 0.43, *p* < 0.001). There is also a discernible negative correlation between perceived discrimination and trait mindfulness (*r* = -0.19, *p* < 0.01). These correlation results are congruent with our theoretical framework and provide preliminary basis basis for subsequent mediation and moderation analyses.

**Table 1:** Means, standard deviations, correlations of all variables

Variables	Mean	SD	1	2	3	4
1. Self-stigma	2.39	0.85	1			
2. Perceived Discrimination	4.07	0.89	0.51***	1		
3. Trait Mindfulness	3.32	0.52	-0.26***	-0.19**	1	
4. WWB	3.73	0.80	-0.57**	-0.56**	0.43***	1

*NotesN* = 316; *p < 0.05, **p < 0.01, ***p < 0.001. WWB = Workplace well-being

### Test of mediating moderation effect

Firstly, we employed Model 4 of the PROCESS plugin, a development by Darlington and Hayes [61], in examining the mediating role of perceived discrimination within the relationship between self-stigma and WWB whilst controlling for gender, age, and the duration of internship. The results, delineated in Table 2, present a significant negative predictive influence of self-stigma on WWB (*β*= -0.56, t= -11.35, *p* < 0.001), thereby confirming *H1*. Moreover, with the inclusion of perceived discrimination as a variable in the model, the direct predictive influence of self-stigma on WWB continues to be significant (*β*= -0.36, t= -4.74, *p* < 0.001). Both the direct influence of self-stigma on WWB and the mediating influence of perceived discrimination exclude zero within the bootstrap 95% confidence intervals (CIs) (refer Table 3). The results suggest that self-stigma significantly predicts WWB in medical students with disabilities, effected not only directly but also through the mediating role of perceived discrimination, thus supporting *H2*. In particular, the direct effect (− 0.34) and mediating effect of perceived discrimination (− 0.12), account for 60.71% and 21.43% of the total effect (− 0.56) respectively.

**Table 2:** Results of mediation model of self-stigma and WWB

DV	IV	*R*	*R*²	F(df)	β	*t*
**WWB**		0.61	0.40	43.76(5)		
	Gender				0.48	5.13**
	Age				0.08	1.01
	Tenure				0.25	2.92**
	Self-stigma				-0.56	-11.35***
**Perceived Discrimination**		0.70	0.50	23.38(5)		
	Gender				-0.01	-0.14
	Age				0.14	1.49
	Tenure				-0.10	-1.15
	Self-stigma				0.48	8.45***
**WWB**		0.69	0.49	54.36(6)		
	Gender				0.47	5.56**
	Age				0.18	1.79
	Tenure				0.20	2.65**
	Perceived Discrimination				-0.35	-8.23***
	Self-stigma				-0.36	-4.74***

*NotesN* = 316. *p < 0.05, **p < 0.01, ***p < 0.001. DV = Dependent variable; IV = Independent variable. WWB = Workplace well-being

**Table 3:** Results of total effects, direct effects, and mediation effects

	Effect size	SE	LLCI	ULCI	Relative effect size
Total effect	-0.56	0.03	-0.64	-0.47	
Direct effect	-0.34	0.04	-0.45	-0.27	60.71%
Mediation effect	-0.12	0.02	-0.23	-0.10	21.43%

*Notes* Bootstrap sample size = 5000. LLCI = Bias corrected lower limit confidence interval; ULCI = Bias corrected upper limit confidence interval

### Test of moderated mediation effect

**Fig. 2:**
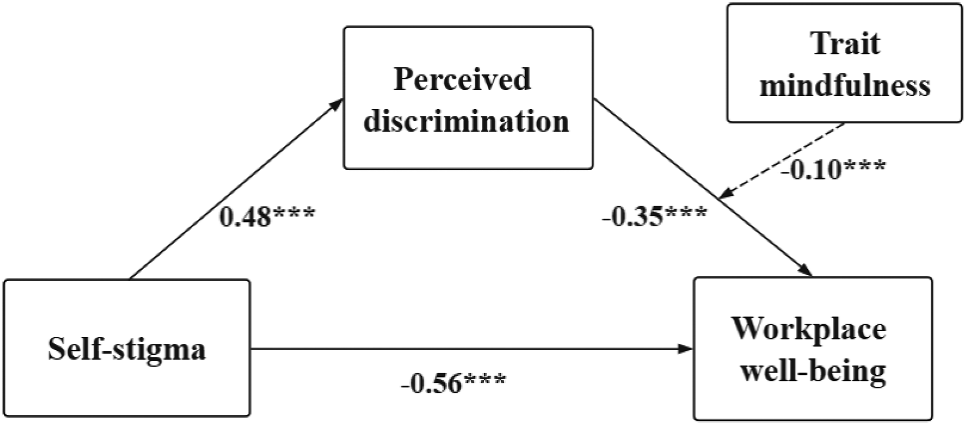
Results of the model

**Fig. 3:**
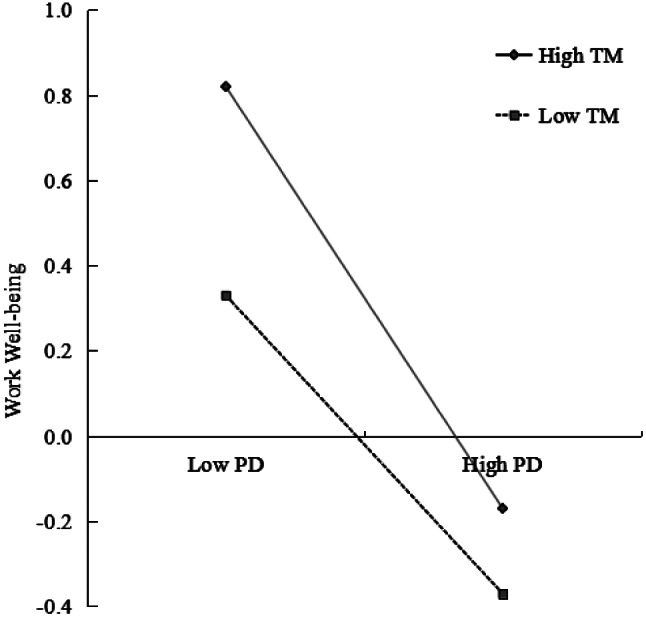
The moderating effect of trait mindfulness on the association between perceived discrimination and WWB

A moderated mediation model was assessed applying Model 14 of the PROCESS plugin [62], while adjusting for gender, age, and duration of the internship. The results (see Table 4) reveal that when incorporating trait mindfulness into the model, the interaction term (PD × TM) significantly predicts WWB (*β*= -0.10, t = -3.76, *p* < 0.001). This implies that trait mindfulness functions predictively within the mediating mechanism for the Self-stigma→Perceived Discrimination→WWB relationship, corroborating *H3*. These data illustratively confirm the validity of the posited structural equation model (refer Fig. 2), revealing: (1) A notable negative correlation between self-stigma and WWB exists among disabled medical students; (2) Perceived discrimination partially mediates the correlation between self-stigma and WWB; (3) Trait mindfulness moderates the latter segment of the mediating pathway. For a deeper insight into the interaction effect between perceived discrimination and trait mindfulness, a graphical representation for simple slope analysis was created based upon trait mindfulness scores (see Fig. 3). The results indicated in Table 5, show that at lowered levels of trait mindfulness (M-1 SD), perceived discrimination significantly and negatively predicts WWB (simple slope = -0.45, t = -6.47, *p* < 0.001). With greater levels of trait mindfulness (M + 1 SD), a significant predictive effect of perceived discrimination on WWB remains (simple slope = -0.15, t =-2.52, *p* < 0.05); but, this effect weakens, suggesting a diminishing predictive influence of perceived discrimination on WWB as trait mindfulness levels rise. In essence, an increase in the trait mindfulness levels among disabled medical students correlates with a consequent improvement in their WWB.

**Table 4:** Results of moderated mediation model

DV	IV	*R*	*R*²	F(df)	β	*t*
**WWB**		0.76	0.59	59.62(8)		
	Gender				0.49	6.30**
	Age				0.06	1.10
	Tenure				0.13	2.09
	Self-stigma				-0.32	-7.76***
	Perceived Discrimination				-0.31	-7.72***
	Trait Mindfulness				-0.33	8.47***
	PD×TM				-0.10	-3.76***

*NotesN* = 316. *p < 0.05, **p < 0.01, ***p < 0.001. DV = dependent variable; IV = independent variable. WWB = Workplace well-being, PD = Perceived Discrimination, TM = Trait Mindfulness

**Table 5:** Bootstrap tests with moderated mediation effects

Trait mindfulness levels	Effect size	SE	LLCI	ULCI
-6.47(M-1SD)	-0.45	0.03	-0.56	-0.40
-5.1(M)	-0.30	0.02	-0.43	-0.27
-2.52(M + 1SD)	-0.15	0.05	-0.27	-0.03

*Notes* Bootstrap sample size = 5000. LLCI = Bias corrected lower limit confidence interval; ULCI = Bias corrected upper limit confidence interval

## Discussion

Drawing on the CCT, this study investigated the between self-stigma and WWB and the moderating effect trait mindfulness on these relation among medical students with physical disabilities. Our findings indicate that self-stigma significantly negatively correlates with WWB, with perceived discrimination mediating this relationship. Furthermore, trait mindfulness moderated the impact of perceived discrimination on WWB, supporting our hypotheses.

First, this investigation scrutinizes the impact of self-stigma on WWB through a lens of cognitive consistency, thereby enriching the theoretical perspective underpinning WWB research. Predominantly, extant studies have hinged upon the self-determination theory or have integrated individual levels to probe into the genesis and content architecture of WWB [24, 63]. Nonetheless, research illuminating the predictors of WWB from a cognitive standpoint is notably limited. From a cognitive perspective, WWB with transient characteristics epitomizes a relatively stable psychological state that individuals forge based on their enduring appraisal of their work. This state may be transiently influenced by others’ evaluations and emotional occurrences (e.g., internalization of stigmas and nuanced perceptions of discrimination), as well as by particular individual traits, such as trait mindfulness [10].

Second, we employ the theory of cognitive dissonance to elucidate the mediating role of perceived discrimination triggered by self-stigma [64]. The rationale is that when medical students with physical disabilities amplify their perceptions of discriminatory cues in the workplace, or even self-discriminate due to self-stigma, a conflict arises between their intuitive expectation that ‘work promotes self-worth and social integration’ and the negative ‘reality’ of their professional competence and work contributions being devalued. This leads to cognitive dissonance. Driven by aversive consequences [65], to alleviate the discomfort arising from this dissonance, these students further convince themselves of their own culpability, engaging in self-doubt and self-pity that are detrimental to their WWB. This finding furnish novel empirical insights into the study of WWB among disabled medical students.

Third, research into the interplay between perceived discrimination and trait mindfulness has uncovered the moderating role of mindfulness in mitigating attentional biases in medical students with disabilities. This enriches our understanding of the limits within which perceived discrimination operates. Special populations, such as those with high anxiety, aggressiveness, or low self-esteem, often exhibit attentional biases, as robustly supported by existing evidence [66]. However, the majority of research on these attentional tendencies within special populations centered on individuals with psychological disorder, overlooking the physically disabled. Our research suggests that medical students with disabilities potentially exhibit attentional biases akin to those in other distinct groups. These individuals are frequently more receptive to microaggressions or ‘sensitive’ cues in their work environment, and they grapple with challenges in diverting their attention away from negative stimuli and overly ‘self-focused’ cognition. Such attentional biases, rooted in the perceived discrimination, might be a important factor distancing these disabled students from WWB. While, as a constructive meta-cognitive tool, trait mindfulness intervenes by breaking these attentional biases and freeing this demographic from ‘self-absorption.’

### Practical implications

The current study has demonstrated that self-stigma not only directly undermines WWB of medical students with physical disabilities but also indirectly affects it through perceived discrimination as a mediating variable. Consequently, multifaceted support from educational institutions is crucial in alleviating the effects of self-stigma and perceived discrimination. It is important for medical colleges to recognize that traditional pedagogical approaches, which primarily focus on special education, do not meet the complex needs for psychological rehabilitation and professional development during medical internships and formal practice. Medical colleges should enhance efforts to support the psychophysical well-being of these students. For instance, the enrollment process should include comprehensive health assessments and systematic documentation to provide holistic guidance that integrates medical, rehabilitative, and educational interventions. Recognizing a nurturing support system can boost their sense of security and emotional attachment, effectively reducing self-stigma and limiting tendencies toward excessive introspection and self-discrimination.

Furthermore, research findings indicate that trait mindfulness significantly moderates the negative impact of perceived discrimination on WWB. Numerous clinical studies confirm the therapeutic advantages of trait mindfulness in the psychological rehabilitation of individuals with disabilities [67, 68]. Practically, medical students with physical disabilities, transitioning to medical practitioners, require a deep sense of self-identification and emotional resilience to manage the inevitable challenges in medical practice. The enduring nature of some physical disabilities highlights the importance of trait mindfulness as an effective psychological intervention for those experiencing the effects of internalized stigma and perceived discrimination. More importantly, the ‘decentering’ aspect of mindfulness training closely aligns with the collectivist ethos prevalent in settings such as medical colleges and healthcare facilities in Eastern cultures. Influenced by Confucian principles, Eastern communities generally regard disabilities as unequivocal realities, acknowledging the futility of denial and choosing to confront challenges. Consequently, medical schools and healthcare training institutions can customize mindfulness training for this demographic, incorporating approaches like mindfulness-based cognitive therapy and meditation exercises. After receiving such training, disabled medical students who are immersed in Eastern cultures are likely to perceive their impairments objectively. This can foster enhanced self-transcendence and improve their positive cognitive experience at work.

### Strength and limitations

This research concentrates on a crucial yet underrepresented group within the domain of higher medical education: medical students with physical disabilities. The examination of this group is both timely and relevant, considering the ongoing societal and educational reforms. This study employs the CCT to elucidate the intricate psychological interactions of these students. It delves into the interplay between self-stigma, perceived discrimination, trait mindfulness, and WWB. By exploring the nexus of disability, stigma, and well-being within a specific professional milieu, this research enriches the discourse concerning the integration of medical students with physical disabilities into broader societal.

Several limitations need to be acknowledged. First, there are limitations associated with the use of cross-sectional data. Although the data for this study were drawn from the same set of subjects, it is challenging to make conclusive causal inferences based solely on observational data. To address this, future studies might consider implementing experimental designs, qualitative methodologies, or leveraging cross-reports and multi-period lag techniques. Such approaches would facilitate a more robust examination of causal relationships and offer stronger empirical support. Second, concerns arise regarding the representativeness of the study’s participants. The distinct nature of the subject group, coupled with sampling challenges, resulted in a modest sample size of 316. Subsequent studies should aim to enlarge this sample to better ascertain the model’s applicability. Third, subjects with diverse disability types and levels might have varied perceptions concerning self-stigma, perceived discrimination, trait mindfulness, and WWB. A more detailed comparative analysis is warranted to unpack the nuances of WWB among these students based on the severity and nature of their disabilities.

## Conclusions

Guided by the CCT, we constructed a conceptual model to explore perceived discrimination arising from self-stigma among medical students with physical disabilities, and its impact on their WWB. Given the detrimental link between perceived discrimination and positive work experiences, exploring psychological mechanisms to alleviate such negative outcomes could hold potential benefits for this demographic trapped by self-stigma. Consistent with our hypothesis, trait mindfulness appears to blunt the association between the perception of discrimination and WWB. Consequently, enhancing trait mindfulness in medical students who are contending with negative cognitive reactions due to perceived discrimination can significantly lessen its adverse impact on WWB. This approach offers crucial insights for educational and psychological interventions aimed at medical students with disabilities. Additionally, our findings provide strategic recommendations for medical schools and healthcare institutions, advocating the promotion of mindfulness practices and awareness to foster an inclusive and supportive environment for medical students with disabilities.

## Data Availability

The datasets generated and/or analyzed during the current study are not publicly available due to information that could compromise the privacy of research participants, but are available from the corresponding author on reasonable request.

## Abbreviations

*WWB*: Workplace well-being
*CCT*: Cognitive consistency theory
*ISMIS*: Mental Illness Scale
*DPQ*: Discrimination Perception Questionnaire
*WWBS*: Workplace Well-being Sub-scale
*MAAS*: Mindful Attention Awareness Scale
*SWB*: Subjective well-being
*EWBS*: Employee Well-being Scale
